# Supplementary material for: Prediction of clinical trial enrollment rates
Source: PLoS One. 2022 Feb 24;17(2):e0263193. doi: 10.1371/journal.pone.0263193 (PMC8870517; doi:10.1371/journal.pone.0263193)
Supplement: S1 File — (PDF) [file pone.0263193.s001.pdf]

Supplemental Information on mathematical justification for domain adaptation

Standard machine learning validation protocols such as cross-validation or holdout splits assume that the samples in the training set and the validation set are i.i.d (independent and identically distributed). However, sometimes this assumption is violated. Since the goal of machine learning is to produce a classifier that performs well on future samples, a procedure to correct for the difference in the sampling distributions may be warranted. This area of research is called *domain adaptation* in the machine learning literature [30–33].

The goal of supervised learning is to minimize the *risk* for future predictions. The risk is defined as

$$R = \mathbf{E}_f \left[L(h(X), Y) \right] \tag{6}$$

where X is a feature vector, Y is the target for prediction, h is the classification function, and L is the loss function. The random variables X and Y are distributed according to the joint distribution $f(x, y)$. The risk is the expected loss for a classification function over a particular distribution of data. The finite sample estimator for the risk is

$$\hat{R} = \frac{1}{n} \sum_{i=1}^{n} L(h(x_i), y_i) \tag{7}$$

where n is the sample size.

Domain adaptation may be necessary when the joint distribution of the data, $f(x, y)$, is different in the training set and the validation set. In other words, the training set is drawn from a distribution $f_{\mathcal{S}}$ and the validation set is drawn from a distribution $f_{\mathcal{T}}$. The distributions $f_{\mathcal{S}}$ and $f_{\mathcal{T}}$ are referred to as the *source domain* and the *target domain*. If the source distribution and target distribution are different, then the target risk, $R^{\mathcal{T}}$, can be related to the source risk as follows [31, 33]:

$$
\begin{aligned}
R^{\mathcal{T}} &= \mathbf{E}_{f_{\mathcal{T}}} \left[L(h(X), Y) \right] && (8) \\
&= \sum_y \int_x L(h(x), y) f_{\mathcal{T}}(x, y) dx && (9) \\
&= \sum_y \int_x L(h(x), y) \frac{f_{\mathcal{T}}(x, y)}{f_{\mathcal{S}}(x, y)} f_{\mathcal{S}}(x, y) dx && (10) \\
&= \mathbf{E}_{f_{\mathcal{S}}} \left[\frac{f_{\mathcal{T}}(X, Y)}{f_{\mathcal{S}}(X, Y)} L(h(X), Y) \right] && (11)
\end{aligned}
$$

The ratio $f_{\mathcal{T}}/f_{\mathcal{S}}$ is called the importance weight, w.

$$w(x, y) = \frac{f_{\mathcal{T}}(x, y)}{f_{\mathcal{S}}(x, y)} \tag{12}$$

Observations (x, y) with large weights are relatively more likely to occur in the testing data than the training data. In practice, the distributions $f_{\mathcal{T}}$ and $f_{\mathcal{S}}$ are unknown and must be estimated. There are various methods in the literature for estimating the importance weights. Once the importance weights are estimated, they can be provided to a classification algorithm together with the training data for training the model. Many predictive modeling algorithms such as support vector machines, random forests, and logistic regression can accept weights for the training samples.

For our data set, the importance weights can be directly computed by adding an auxiliary random variable, T, the study duration, to the joint distributions $f_{\mathcal{S}}$ and $f_{\mathcal{T}}$. Consider the following modification of Eqs 8–11, which explicitly incorporates the auxiliary variable:

$$\begin{aligned}
R^{\mathcal{T}} &= \mathbf{E}_{f_{\mathcal{T}}}\left[L(h(X), Y)\right] && (13)\\
&= \sum_y \int_x \int_0^\infty L(h(x), y) f_{\mathcal{T}}(x, y, t) dt\, dx && (14)\\
&= \sum_y \int_x \int_0^\infty L(h(x), y) \frac{f_{\mathcal{T}}(x, y, t)}{f_{\mathcal{S}}(x, y, t)} f_{\mathcal{S}}(x, y, t) dt\, dx && (15)\\
&= \mathbf{E}_{f_{\mathcal{S}}}\left[\frac{f_{\mathcal{T}}(X, Y, T)}{f_{\mathcal{S}}(X, Y, T)} L(h(X), T)\right] && (16)
\end{aligned}$$

Note that the classifier h does not use the study duration in its classification rule. We assume that $f_{\mathcal{T}}$ is a truncated form of $f_{\mathcal{S}}$ according to the truncation $T < \tau$, where τ is the maximum possible study duration in the validation set:

$$f_{\mathcal{T}}(x, y, t) = f_{\mathcal{S}}(x, y, t \,|\, t < \tau) \tag{17}$$

Then

$$f_{\mathcal{T}}(x, y, t) = \frac{1}{Z} f_{\mathcal{S}}(x, y, t) I(t < \tau) \tag{18}$$

where Z is a normalization constant:

$$Z = \sum_y \int_x \int_0^\tau f_{\mathcal{S}}(x, y, t) dt\, dx \tag{19}$$

So the importance weight becomes

$$w(x, y, t) = \frac{f_{\mathcal{T}}(x, y, t)}{f_{\mathcal{S}}(x, y, t)} = \frac{1}{Z} I(t < \tau) \tag{20}$$

and the risk estimator becomes

$$\hat{R}_{\mathcal{T}} = \frac{1}{nZ} \sum_{i=1}^{n} I(t_i < \tau) L(h(x_i), y_i) \tag{21}$$

For the purposes of optimizing the classifier h, the constant factor $1/Z$ can be ignored, so the sample importance weights can be defined by $w_i = I(t_i < \tau)$.

Supplemental Fig S1

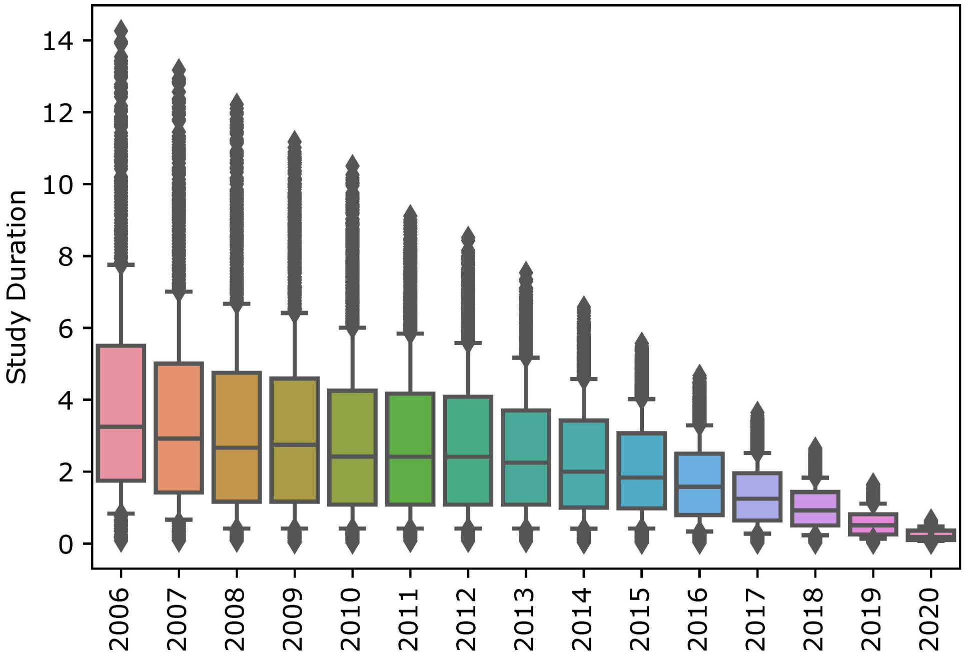

Fig S1. The distribution of clinical trial durations, conditioned on the year of the trial start date. The box whiskers are drawn at the 10th and 90th percentiles of the empirical distributions.

Supplemental Fig S2

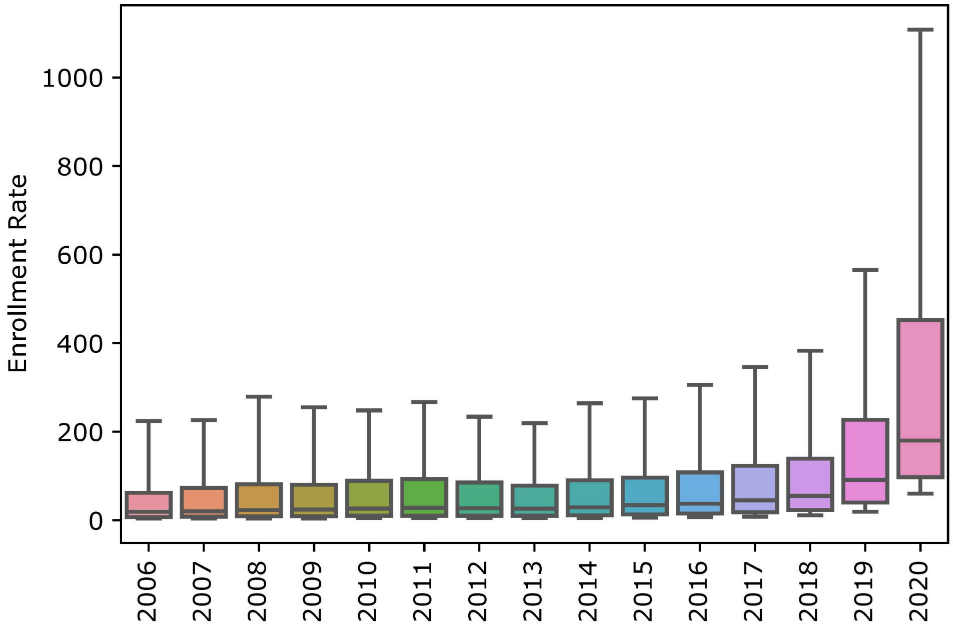

Fig S2. The distribution of enrollment rates, conditioned on the year of the trial start date. The box whiskers are drawn at the 10th and 90th percentiles of the empirical distributions. The enrollment rate distributions are heavy tailed, so outlier points are omitted from the plot to keep the scale legible.

Supplemental Fig S3

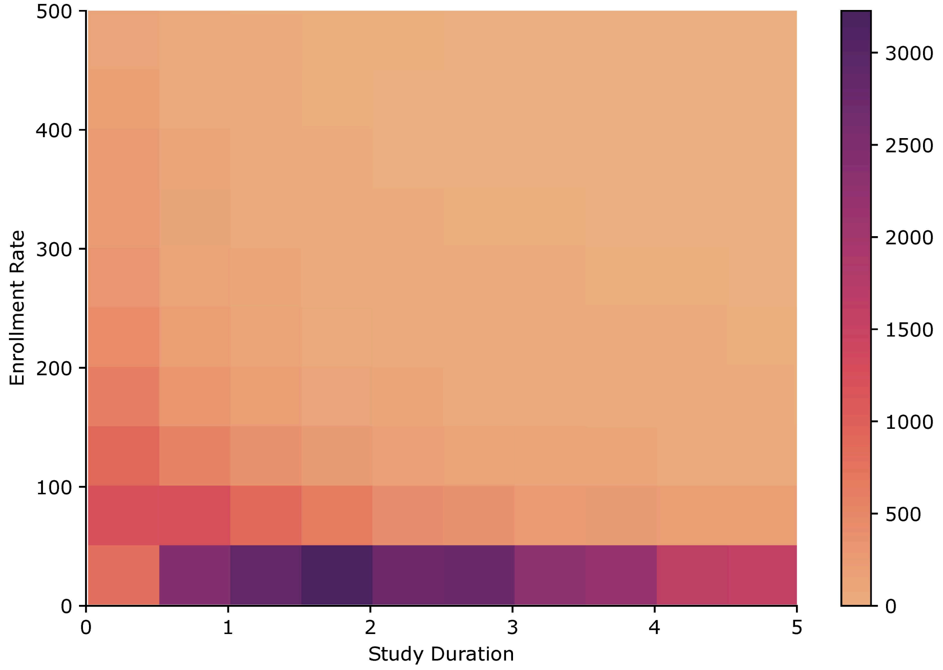

Fig S3. A 2D histogram of enrollment rate vs study duration. There is an inverse relationship between enrollment rate and study duration.

Supplemental Table S1

Model	Parameter	Values
KNN	# neighbors	5, 10, 20, 50, 100
Elastic Net	C	1×10^{-4}, 7.7×10^{-4}, 6.0×10^{-3}, 4.6×10^{-2}, 0.36, 2.78, 21.5, 167, 1290, 1000
	l1-ratio	.1, .2, .5, .7, .9, .95, .99, 1
Random Forest	# trees	200, 400, 600, 800
	min samples per leaf	1, 2, 5, 10
SVM	degree	1, 2, 3, 4
	C	10^{-4}, 10^{-3}, 10^{-2}, 10^{-1}, 1, 10, 100, 1000, 10000

Table S1. Parameterization of classifiers: the values for the hyper-parameters that were evaluated during the grid search for model tuning. The logistic classifier and the dummy model do not have hyper-parameters to tune.

Supplemental Table S2

Model	Training Set	AUC	Recall	Precision	Accuracy
dummy	complete	0.500 (0.000)	0.333 (0.000)	0.157 (0.021)	0.471 (0.062)
dummy	mesh	0.500 (0.000)	0.333 (0.000)	0.157 (0.021)	0.471 (0.062)
dummy	population	0.500 (0.000)	0.333 (0.000)	0.157 (0.021)	0.471 (0.062)
dummy	study center	0.500 (0.000)	0.333 (0.000)	0.157 (0.021)	0.471 (0.062)
dummy	study design	0.500 (0.000)	0.333 (0.000)	0.157 (0.021)	0.471 (0.062)
knn	complete	0.750 (0.021)	0.552 (0.019)	0.557 (0.026)	0.591 (0.047)
knn	mesh	0.642 (0.029)	0.440 (0.026)	0.454 (0.023)	0.489 (0.041)
knn	population	0.578 (0.011)	0.392 (0.013)	0.422 (0.027)	0.477 (0.050)
knn	study center	0.607 (0.015)	0.415 (0.014)	0.450 (0.033)	0.498 (0.055)
knn	study design	0.746 (0.021)	0.539 (0.015)	0.553 (0.030)	0.587 (0.049)
logistic classifier	complete	0.743 (0.011)	0.557 (0.010)	0.557 (0.014)	0.593 (0.034)
logistic classifier	mesh	0.662 (0.019)	0.456 (0.020)	0.468 (0.023)	0.509 (0.039)
logistic classifier	population	0.539 (0.014)	0.371 (0.007)	0.350 (0.036)	0.486 (0.069)
logistic classifier	study center	0.564 (0.013)	0.377 (0.010)	0.366 (0.048)	0.490 (0.065)
logistic classifier	study design	0.743 (0.023)	0.540 (0.015)	0.544 (0.027)	0.586 (0.049)
elastic net	complete	0.772 (0.018)	0.571 (0.014)	0.575 (0.023)	0.613 (0.042)
elastic net	mesh	0.674 (0.022)	0.455 (0.019)	0.475 (0.022)	0.516 (0.041)
elastic net	population	0.539 (0.014)	0.371 (0.007)	0.350 (0.036)	0.486 (0.069)
elastic net	study center	0.565 (0.013)	0.378 (0.008)	0.375 (0.058)	0.491 (0.064)
elastic net	study design	0.743 (0.023)	0.540 (0.016)	0.544 (0.027)	0.586 (0.049)
random forest	complete	0.776 (0.028)	0.573 (0.023)	0.585 (0.037)	0.617 (0.052)
random forest	mesh	0.675 (0.022)	0.472 (0.023)	0.486 (0.023)	0.520 (0.041)
random forest	population	0.594 (0.012)	0.399 (0.015)	0.435 (0.030)	0.492 (0.054)
random forest	study center	0.624 (0.013)	0.435 (0.013)	0.459 (0.024)	0.508 (0.049)
random forest	study design	0.760 (0.021)	0.554 (0.014)	0.566 (0.025)	0.601 (0.047)
svm	complete	0.805 (0.016)	0.578 (0.016)	0.589 (0.025)	0.615 (0.043)
svm	mesh	0.677 (0.024)	0.454 (0.019)	0.479 (0.024)	0.510 (0.042)
svm*	population	–	–	–	–
svm*	study center	–	–	–	–
svm	study design	0.772 (0.023)	0.548 (0.016)	0.566 (0.029)	0.597 (0.049)

Table S2. The performance of various classifiers on various feature sets, *mean* (*standard deviation*). *Did not finish training in the allotted run time (96 hrs).

Supplemental Table S3

Model	Training Set	AUC	Recall	Precision	Accuracy
dummy	complete	0.500 (0.000)	0.333 (0.000)	0.157 (0.021)	0.471 (0.062)
dummy	mesh	0.500 (0.000)	0.333 (0.000)	0.157 (0.021)	0.471 (0.062)
knn	complete	0.751 (0.021)	0.548 (0.016)	0.556 (0.031)	0.594 (0.050)
knn	mesh	0.578 (0.037)	0.384 (0.028)	0.430 (0.039)	0.457 (0.086)
logistic classifier	complete	0.760 (0.023)	0.557 (0.018)	0.558 (0.027)	0.601 (0.048)
logistic classifier	mesh	0.618 (0.029)	0.382 (0.013)	0.443 (0.026)	0.487 (0.056)
elastic net	complete	0.760 (0.024)	0.557 (0.019)	0.559 (0.029)	0.601 (0.050)
elastic net	mesh	0.618 (0.029)	0.381 (0.013)	0.443 (0.027)	0.487 (0.056)
random forest	complete	0.775 (0.018)	0.568 (0.011)	0.582 (0.025)	0.615 (0.043)
random forest	mesh	0.616 (0.030)	0.387 (0.015)	0.437 (0.026)	0.485 (0.055)
svm*	complete	0.803 (0.021)	0.568 (0.016)	0.583 (0.027)	0.619 (0.047)
svm	mesh	0.609 (0.029)	0.385 (0.014)	0.433 (0.024)	0.482 (0.055)

Table S3. The performance of various classifiers using the reduced top MeSH feature set (MeSH terms that appeared at least 200 times). *complete* refer to the reduced MeSH set + other data module and *MeSH-only* refer to the reduced MeSH set by itself, *mean* (*standard deviation*). *Metrics are based on partial results. Nested cross-validation folds were run in parallel, but a few did not finish within the allotted run time (96 hours).
