# Supplementary material for: Prediction of clinical trial enrollment rates
Source: PLoS One. 2022 Feb 24;17(2):e0263193. doi: 10.1371/journal.pone.0263193 (PMC8870517; doi:10.1371/journal.pone.0263193)
Supplement: Attachment — Submitted filename: response_to_review.pdf [file pone.0263193.s002.pdf]

Response to Review for Prediction of Clinical Trial Enrollment Rates

January 6, 2022

1 Overall Response to Review

We thank the reviewers for the constructive comments and the editor of organizing the review process. We took the reviewers advice and made the following changes to the paper:

- Strengthened and broadened the literature review
- Added discussion of the contribution and limitation of the current study
- Modified introduction to highlight the objective, scope, and organization of the paper.
- Other minor changes for clarity according to the reviewers suggestions.

We believe our revision had improved the overall quality of the paper, and we thank the editors and reviewers for the opportunity for the revision.

In the section below, we provide a point by point response to the reviewers comments.

2 Sepecific Response to Review

Note: Reviewers comments are marked in black, the authors' responses are marked in blue.

1. Is the manuscript technically sound, and do the data support the conclusions?

The manuscript must describe a technically sound piece of scientific research with data that supports the conclusions. Experiments must have been conducted rigorously, with appropriate controls, replication, and sample sizes. The conclusions must be drawn appropriately based on the data presented.

Reviewer 1: Yes

Reviewer 2: Yes

> We thank the reviewers for the positive feedback regarding the technical quality of the paper.

2. Has the statistical analysis been performed appropriately and rigorously?

Reviewer 1: Yes

Reviewer 2: Yes

> We thank the reviewers for the positive feedback regarding the statistical analysis.

3. Have the authors made all data underlying the findings in their manuscript fully available?

The PLOS Data policy requires authors to make all data underlying the findings described in their manuscript fully available without restriction, with rare exception (please refer to the Data Availability Statement in the manuscript PDF file). The data should be provided as part of the manuscript or its supporting information, or deposited to a public repository. For example, in addition to summary statistics, the data points behind means, medians and variance measures should be available. If there are restrictions on publicly sharing data—e.g. participant privacy or use of data from a third party—those must be specified.

Reviewer 1: Yes

Reviewer 2: No

> All data used in this study is publicly available, we modified the methods section with more detailed descriptions regarding how the data can be obtained. Specifically, the following description for the three data sources used in the study: (1) We downloaded the XML formatted clinical trial records for all studies on the website on October 28, 2020 from `https://clinicaltrials.gov/ct2/resources/download.`; (2) Statistics on metropolitan and micropolitan area populations were obtained from the U.S. Census Bureau[10] from the url `https://www2.census.gov/programs-surveys/popest/tables/2010-2018/state/totals/PEP_2018_PEPANNRES.zip`. and (3) The (Nature Index score) data can be obtained from `https://www.natureindex.`

com/annual-tables/2018/institution/all/all and selecting *United States of America* as the region or country.

4. Is the manuscript presented in an intelligible fashion and written in standard English?

PLOS ONE does not copyedit accepted manuscripts, so the language in submitted articles must be clear, correct, and unambiguous. Any typographical or grammatical errors should be corrected at revision, so please note any specific errors here.

Reviewer 1: No

Reviewer 2: Yes

> We have modified the mauscript throughout, specifically in the introduction and discussion section, and improved the clarity. See attached manuscript with track changes and specific response to reviewer 1's comments below.

5. Review Comments to the Author

Reviewer 1: The Research Paper needs the following Major Revisions and is Subject for re-review, and after re-review, the final decision for the paper will be done:

a. Abstract- In the last lines, highlight regarding experimental analysis.

> We edited the abstract according to the reviewers suggestion. Specifically, the following sentenses were added to the end of the abstract: "Taking advantage of a nested time series cross-validation design, our models resulted in good predictive performance that is generalizable to future data and stable over time. Moreover, information content analysis revealed the study design related features to be the most informative feature type regarding enrollment. Compared to the performance of models built with all features, the performance of models built with study design related features is only marginally worse ($AUC = 0.78 \pm 0.03$ vs. $AUC = 0.76 \pm 0.02$)."

b. Introduction should be more broad and should cover more towards Problem Definition and Scope.

> We restructured the introduction and added more content following the suggestion of the reviewer.

To give a more complete picture of the enrollment challenge for clinical trials, we dedicate the first paragraph to describe the extend and severity of the problem. The second paragraph describes the identified barriers and existing efforts to improve enrollment for on going clinical trials.

Then, we define the problem we are tackling in the current study and our scope in the third paragraph of the introduction section. Specifically, we

stated “The key challenge is the cost incurred by launching trials that are unlikely to meet their enrollment goal. The current study aims to minimize this cost by building models to predict the enrollment rate prior to the start of the trial. Accurate enrollment rate prediction model provides valuable information for the cost benefit analysis for launching future clinical trials and is a first step towards a decision support system for clinical trials.

Add Objectives of the paper at the end of Introduction

> To highlight the objectives of the paper, we modified the last paragraph of the previous version of the paper (now the third to the last paragraph) as the following:“The main objective of the current study is to build models for clinical trial enrollment prediction using available information prior to the initiation of the trial. To address the above mentioned gaps in the literature, we specified four sub-objectives and briefly describe the strategies to achieve these sub-objectives: (1) To build generalizable models over different institutions and organizations that produce robust and stable performance over time: we leveraged a data set consisting of 46,724 U.S. based clinical trials from 1990 to 2020 extracted from *ClincalTrials.gov*, covering a broad trial population. (2) To examine a larger variety of features for enrollment prediction and improve predictive performance: we constructed a large number of candidate predictors regarding various aspects of individual clinical trials, including characteristics regarding the targeted trial population, the institution responsible for recruitment, the medical domain of the trial, and the design of the trial. (3) To construct predictive models that do not rely on estimated enrollment rate and produce good performances: We applied a variety of state-of-the-art supervised learning methods to build predictive models for enrollment rate prediction. (4) To identify the feature type that is the most informative for enrollment rate prediction: we compared the information content for enrollment rate prediction among models constructed with different feature types.”

Add Organization of the paper

> We have added the organization of the paper to the end of the introduction (second to the last paragraph), as the following: “The organization of the manuscript is as the following: In the methods section, we first describe the data acquisition and processing procedures. We then introduce the design of our analysis, including the time series cross-validation protocol for model selection and performance estimation, and the comparison of information content in various feature types. We also introduced the metrics for predictive performance examined. In the results section, we present the

performance of various models for predicting enrollment rate. In the discussion section, we highlight the contribution of the study, note the limitations, and point to potential future work."

c. Add some Literature review to this paper with min 4-10 Papers.

> Following the reviewers' suggestion, we restructured our prior literature review paragraph in the introduction section, expanded on it, and gave it more context, and included additional studies that are relevant. Specifically, the first paragraph introduced the literature describing the severity of the clinical trial enrollment problem. The second paragraph introduced literature regarding efforts for improving enrollment for on-going clinical trials. The forth paragraph introduced literature regarding the prediction of enrollment, including two broad categories of research, parametric models for enrollment prediction and factors influencing enrollment. We also discussed the gap in the enrollment prediction literature in the forth paragraph. The current literature review contains 20 papers, with 10 additional papers.

d. Add some case study based discussion to the paper.

> In the new subsection "Limitations and Future Work" of the discussion section, we highlighted several prior studies using the *ClinicalTrial.gov* data for a variety of applications and discussed how the methodologies employed in these studies can be incorperated into future work for predicting enrollment rate. Specifically, "Natural Language Processing methods applied to the free-text data from *ClinicalTrials.gov* has been explored for various applications including construction of knowledge base for eligibility criterion [6], illustration of relationships among multiple clinical trials [2], and literature mining for trends and prevalence of clinical instruments [11]. However, using Natural Language Processing methods for feature construction for predictive task using the *ClinicalTrials.gov* data is an untapped area. We expect the combination of free-text based features and structured data could improve the predictive performance for enrollment rate, as demonstrated in prior studies in other data domain [7, 5, 1].", and, "Potential external sources of information include information related to the principle investigator of the trial([4] showed several possible feature constructions related to this), the prevalence of the disease or condition in question near the recruitment location ([9] demonstrated a way to construct these variables), and the characteristics of the population (age, gender, education, insurance status, etc., can be obtained from census data[10]) near the recruitment location."

e. Add conclusion and future scope to the paper

> We modified the last paragraph of the discussion section and made it

into a conclusion section. Specifically, the conclusion section is the following: “The current study emperically demonstrated the feasibility of enrollment prediction prior to the initiation of clinical trials. To the best of our knowledge, this is the first study that employs machine learning methods for enrollment rate prediction based on a large and comprehensive sample of U.S.-based clinical trials. This study is the first step towards a data-driven decision support system for assessing whether a proposed clinical trial would likely meet its enrollment goal.”

> We restructured the discussion section to emphasize future work by adding a subsection named “Limitations and Future Work”. We highlighted two main source of additional data can be included as features when constructing the next generation models for enrollment rate prediction.

f. Addition of min 5-10 Latest references of 2021 cum 2022 are recommended to th epaper.

> Following the reviewers’ suggestion, we included 12 studies related to the current study published in 2021.

Reviewer 2: This article performs the clinical examination on 4,636 candidate predictors based on data collected by ClinicalTrials.gov and external sources for enrollment rate prediction using various state-of-the-art machine learning methods.This article studied well and may be considered for publication after addressing the below:

1. Highlight the contributions in the Introduction.

> We added contributions as the last paragraph of the introduction section as the following: “The main contribution of the current study is that we constructed generalizable models for clinical trials enrollment prediction with good predictive performance and are stable over time. Our results empirically demonstrated the feasibility of predicting enrollment rate prior to the initiation of clinical trials. Thus, this study forms the basis for data-driven decision support systems to assess whether proposed clinical trials would likely meet their enrollment goal.”

2. Provide the numbering for subsections of the article.

> To our knowledge, the journal format does not use numbering for sections and subsections. Personally, we prefer numbered sections as well.

3. PRovide the literature study.

> Following the reviewers’ suggestion, we restructed our prior literature review paragraph in the introduction section, expanded on it, and gave it more context, and included additional studies that are relevant. Specifically, the first paragraph introduced the literature describing the severity of the

clinical trial enrollment problem. The second paragraph introduced literature regarding efforts for improving enrollment for on-going clinical trials. The forth paragraph introduced literature regarding the prediction of enrollment, including two broad categories of research, parametric models for enrollment prediction and factors influencing enrollment. We also discussed the gap in the enrollment prediction literature in the forth paragraph.

4. The formulae available in the existing papers must be cited.

> We added citations to the performance metrics to the end of the corresponding subsection, specifically, the following sentence was added: "For an in-depth discussion of the properties of these metrics, please see [3, 8]"

5. Add the conclusion to the article.

> We modified the last paragraph of the discussion section and made it into a conclusion section. Specifically, the conclusion section is the following: "The current study emperically demonstrated the feasibility of enrollment prediction prior to the initiation of clinical trials. To the best of our knowledge, this is the first study that employs machine learning methods for enrollment rate prediction based on a large and comprehensive sample of U.S.-based clinical trials. This study is the first step towards a data-driven decision support system for assessing whether a proposed clinical trial would likely meet its enrollment goal."

6. List the limitations for the proposed work.

> We restructured the discussion section to emphasize the limitations by adding a subsection named "Limitations and Future Work". We highlighted two main limitations and source of additional data can be included to address these limitations as future work.

7. How the authors considered missing values in the datasets?

> The treatment of missing values was previously stated in the last paragraph of the section "Performance estimation and model selection". To avoid confusion and improve clarity, we took the paragraph, made minor modification and placed it under a new subsection "Variable Scaling and Missing Values". Specifically, statements regarding the treatment for missing values are the following:"Variable scaling and treatment for missing values were incorporated into the modeling pipeline, so that scaling and imputation on the validation data are done according to the distribution of the training data. This prevents information leak from the validation data into the model training phase. ... Median imputation was used for the continuous variables, and missing indicator columns were added to retain the missingness information. For the categorical variables with missing data, we added a "missing" level

to the categories for that variable to represent the missingness information."

References

[1] W. Boag, O. Kovaleva, T. H. McCoy, A. Rumshisky, P. Szolovits, and R. H. Perlis. Hard for humans, hard for machines: predicting readmission after psychiatric hospitalization using narrative notes. *Translational psychiatry*, 11(1):1–6, 2021.

[2] J. Du, Q. Wang, J. Wang, P. Ramesh, Y. Xiang, X. Jiang, and C. Tao. Covid-19 trial graph: A linked graph for covid-19 clinical trials. *Journal of the American Medical Informatics Association*, 2021.

[3] R. O. Duda, P. E. Hart, and D. G. Stork. *Pattern classification and scene analysis*, volume 3. Wiley New York, 1973.

[4] L. Fu and C. Aliferis. Using content-based and bibliometric features for machine learning models to predict citation counts in the biomedical literature. *Scientometrics*, 85(1):257–270, 2010.

[5] M. Levis, C. L. Westgate, J. Gui, B. V. Watts, and B. Shiner. Natural language processing of clinical mental health notes may add predictive value to existing suicide risk models. *Psychological medicine*, 51(8):1382–1391, 2021.

[6] H. Liu, Y. Chi, A. Butler, Y. Sun, and C. Weng. A knowledge base of clinical trial eligibility criteria. *Journal of Biomedical Informatics*, 117:103771, 2021.

[7] R. Liu, J. L. Greenstein, S. V. Sarma, and R. L. Winslow. Natural language processing of clinical notes for improved early prediction of septic shock in the icu. In *2019 41st Annual International Conference of the IEEE Engineering in Medicine and Biology Society (EMBC)*, pages 6103–6108. IEEE, 2019.

[8] M. Sokolova and G. Lapalme. A systematic analysis of performance measures for classification tasks. *Information processing & management*, 45(4):427–437, 2009.

[9] B. Spoer, J. Feldman, M. Gofine, S. Levine, A. Wilson, S. Breslin, L. Thorpe, and M. Gourevitch. Health and health determinant metrics for cities: A comparison of county versus city-level data. In *APHA's 2020 VIRTUAL Annual Meeting and Expo (Oct. 24-28)*. American Public Health Association, 2020.

[10] U.S. Census Bureau. Metropolitan and micropolitan statistical areas population totals and components of change: 2010-2019. `https://www.census.gov/data/datasets/time-series/demo/popest/2010s-total-metro-and-micro-statistical-areas.html`. [Online; accessed 10-Dec-2019].

[11] H. Zong, B. Hu, Y. Han, Z. Li, X. Zhang, et al. Prevalence and temporal trends analysis of screening and diagnostic instruments in posttraumatic stress disorder: Text mining study. *JMIR Mental Health*, 8(11):e33599, 2021.
